# Novel Potential Interacting Partners of Fibronectin in Spontaneous Animal Model of Interstitial Cystitis

**DOI:** 10.1371/journal.pone.0051391

**Published:** 2012-12-07

**Authors:** Gudrun Treutlein, Roswitha Dorsch, Kerstin N. Euler, Stefanie M. Hauck, Barbara Amann, Katrin Hartmann, Cornelia A. Deeg

**Affiliations:** 1 Clinic of Small Animal Medicine, Center of Clinical Veterinary Medicine, LMU Munich, München, Germany; 2 Institute of Animal Physiology, Department of Veterinary Sciences, LMU Munich, München, Germany; 3 Research Unit for Protein Science, Helmholtz Zentrum München – German Research Center for Environmental Health (GmbH), Neuherberg, Germany; Auburn University, United States of America

## Abstract

Feline idiopathic cystitis (FIC) is the only spontaneous animal model for human interstitial cystitis (IC), as both possess a distinctive chronical and relapsing character. Underlying pathomechanisms of both diseases are not clearly established yet. We recently detected increased urine fibronectin levels in FIC cases. The purpose of this study was to gain further insight into the pathogenesis by assessing interacting partners of fibronectin in urine of FIC affected cats. Several candidate proteins were identified via immunoprecipitation and mass spectrometry. Considerable changes in FIC conditions compared to physiological expression of co-purified proteins were detected by Western blot and immunohistochemistry. Compared to controls, complement C4a and thioredoxin were present in higher levels in urine of FIC patients whereas loss of signal intensity was detected in FIC affected tissue. Galectin-7 was exclusively detected in urine of FIC cats, pointing to an important role of this molecule in FIC pathogenesis. Moderate physiological signal intensity of galectin-7 in transitional epithelium shifted to distinct expression in transitional epithelium under pathophysiological conditions. I-FABP expression was reduced in urine and urinary bladder tissue of FIC cats. Additionally, transduction molecules of thioredoxin, NF-κB p65 and p38 MAPK, were examined. In FIC affected tissue, colocalization of thioredoxin and NF-κB p65 could be demonstrated compared to absent coexpression of thioredoxin and p38 MAPK. These considerable changes in expression level and pattern point to an important role for co-purified proteins of fibronectin and thioredoxin-regulated signal transduction pathways in FIC pathogenesis. These results could provide a promising starting point for novel therapeutic approaches in the future.

## Introduction

Feline idiopathic cystitis (FIC), a common disease occurring in 55–69% of cats with lower urinary tract signs, is the best spontaneous animal model for human interstitial cystitis (IC), also known as painful bladder syndrome [1]–[2]. FIC represents most of its features such as bladder pain, urgency and nocturia in the absence of any other identifiable pathology such as urinary tract infection or bladder carcinoma [1], [3]. The diagnosis of both IC and FIC can only be made by exclusion of other diseases and confirmed in cystoscopy by characteristic mucosal lesions and hemorrhages [4]–[5]. To the patients’ distress, a causative therapy could not be established so far. Moreover, both diseases are characterized by their chronical and relapsing character [1], [6].

Despite extensive research the etiology of FIC and IC is still unknown. In veterinary as well as human medicine there is a consensus that FIC and IC are multifactorial disease syndromes involving the urinary bladder. FIC is currently considered a disease syndrome of several and possibly interrelated mechanisms involving local bladder abnormalities, abnormalities of the nervous and endocrine system as well as environmental factors as triggers for psychoneuroendocrine dysfunction [7]. There is also evidence that viruses, especially feline Calicivirus (FCV), may play a role at least in some cases of FIC [8]. Regarding human IC different theories for the underlying pathomechanism were hypothesized among which were chronic or subclinical infection, autoimmunity, neurogenic inflammation or bladder urothelial defects affecting bladder permeability [9]–[10]. One field of IC research engaged the protein contents in urine in order to find potential diagnostic markers and to gain new insight into the pathophysiology of this disease [11]–[13]. Recently, we identified two differentially expressed proteins in disease, trefoil factor 2 and fibronectin by comparing the protein profiles in urine of healthy and FIC diseased cats using proteomic approaches [14]–[15]. Fibronectin, a widely expressed high-molecular weight glycoprotein, plays an important role in cell adhesion, migration, growth, differentiation and wound healing and takes part in a wide variety of interactions with numerous proteins, such as heparin, collagen and fibrin [16]–[17]. It is significantly upregulated in urine of cats with FIC, indicating a more important role of fibrosis in the pathogenesis of this disease than previously thought [15].

The goal of this study was to closely characterize the fibronectin interaction network in urine and urinary bladder tissue of cats with FIC with the aim to gain further insight into the pathophysiology of this disease.

## Materials and Methods

### Collection and Preparation of Urine of Healthy and FIC Cats

All samples were collected from privately owned cats examined at the Clinic of Small Animal Medicine, LMU Munich, Germany. A total of 46 urine specimens were collected and processed. This study included two groups: the FIC group (n = 26) and the healthy control group (n = 20). Inclusion criteria for the FIC group were clinical lower urinary tract signs, such as hematuria, stranguria, pollakisuria and periuria and exclusion of other diseases of the lower urinary tract such as urolithiasis, bacterial urinary tract infection and structural abnormalities (anomalies and neoplasia) [15]. To determine eligibility for inclusion in this study group, abdominal ultrasonography and abdominal radiographs, urinalysis including determination of the urine specific gravity, urine dipstick and urine sediment and aerobic urine culture were performed. Cats were excluded if they showed any sign for crystalluria, bacteriuria, urolithiasis, evidence of structural urinary tract abnormality, or if results of bacterial culture of the urine sample were positive. Only FIC cases with concurrent obstruction of the urethra were included in the study. Healthy control cats were evaluated for health care at the Clinic of Small Animal Medicine. Inclusion criteria for the healthy group were no clinical signs of urinary tract disease, no abnormalities on physical examination and an unremarkable urinalysis, including specific gravity, dipstick and sediment on the day of inclusion. Any history of prior urinary tract disease led to exclusion from the healthy control group. All procedures performed on any of the cats participating in the study were medically indicated. No experimental animals were involved. Urine samples were originally collected for purposes of clinical work-up and used in scientific research with permission from the Small Animal Clinic of LMU Munich, Munich, Germany. Owners gave their consent to use the samples.

A total of 26 urine samples from FIC cats and 20 urine samples from healthy control cats collected by means of cystocentesis (FIC n = 21, controls n = 20) or catheterization (FIC n = 5) were included. Cats with FIC were sampled within 24 h after the onset of clinical signs. Immediately after sampling, native urine was subjected to urine analysis (see below). Subsequently, urine samples were centrifuged at 2000 rpm at room temperature (RT) for 5 min) and the protein content of supernatants as well as sediments was quantified (see below). Finally, supernatants were divided into aliquots and immediately stored at –80°C until further processing.

### Collection and Preparation of Urinary Bladder Tissue of Healthy and FIC Cats

Urinary bladders from three cats with obstructive FIC and four cats with a healthy urinary tract were obtained freshly post mortem. Cases with FIC were privately owned and presented as patients at the Clinic of Small Animal Medicine, LMU Munich, Germany. The reason for euthanasia of the cats was unrelated to our study. Control cats with healthy urinary tract were euthanized due to diseases unrelated to our study and without pathologic alteration of the urinary tract. Owners gave permission for the clinical samples to be used scientifically. No experimental animals were involved.

Urinary bladders were extracted in their entirety within 30 min after euthanasia and sections of various regions were prepared. Sections were fixed by immersion in Bouin’s solution (Sigma-Aldrich, Deisenhofen, Germany), dehydrated in a series of alcohols and subsequently embedded in paraffin (Microm International, Walldorf, Germany).

### Urinalysis and Protein Quantification

Prior to centrifugation of urine samples, a hand refractometer was used to determine urine specific gravity. Additionally, urinalysis was performed by means of the semi-quantitative urinalysis sticks (Combur-9 Roche Diagnostics, Grenzach-Wyhlen, Germany) for determination of pH, total protein content as well as concentration of glucose, ketones, bilirubin, urobilinogen, nitrite and blood/erythrocytes. After centrifugation of urine samples (2000 rpm, RT, 5 min) within 30 min after collection, urine sediments were examined microscopically. Protein content in urine supernatants of each cat included in the study was determined by Bradford analysis (Sigma-Aldrich, Deisenhofen, Germany).

### Immunoprecipitation of Protein Complexes in Representative FIC Diseased Urine

For immunoprecipitation of fibronectin containing protein complexes, the urine sample (50 µg) of one FIC cat was incubated with a polyclonal rabbit anti-human fibronectin antibody (5 µg IgG) (ThermoFisher, Bonn, Germany), which according to the manufacturer’s declaration also detects feline fibronectin, in immunoprecipitation buffer (0.05 M Tris, 0.15 M NaCl, 0.2% NP40; pH 7.4) at RT for 1 h. As negative control, the same amount of urine was incubated with purified rabbit serum IgG (5 µg) (Sigma-Aldrich, Deisenhofen, Germany) using identical conditions to detect any unspecific antibody binding. Antibody-bound protein complexes were recovered via binding to protein G-Sepharose beads (GE Healthcare, Freiburg, Germany) in illustra MicroSpin G-50 Columns (GE Healthcare). Therefore, 40 µl protein G-Sepharose beads were washed several times with immunoprecipitation buffer on the columns and subsequently incubated with the urine-antibody-, respectively urine-serum-IgG mixture at 4°C for 1 h with gentle agitation. Afterwards, the Sepharose-bound immunoprecipitates were centrifuged at 0.8 rpm for 2 s followed by several washing steps with immunoprecipitation buffer. Immune complexes of both appendages were eluted from beads into 50 µl of Laemmli buffer (4% SDS, 20% glycerol, 10% 2-mercaptoethanol, 0.004% bromphenol blue, 0.125 M Tris; pH 6.8) by agitation on a bench top shaker (1400 rpm) for 10 min and subsequent heating to 70°C for 10 min. After centrifugation, the supernatant containing the immunoprecipitated proteins was separated by SDS-PAGE (8%) and blotted onto polyvinyldifluoride membranes (PVDF; GE Healthcare, Freiburg, Germany). After blocking for 1 h in 1% polyvinylpyrrolidone in PBS-T (PVP-T; PBS containing 0.05% Tween 20) blots were incubated in a 1∶1000 dilution of polyclonal rabbit anti-human fibronectin antibody (ThermoFisher) overnight at 4°C followed by detection of binding by a 1∶1500 dilution of HRP-conjugated goat anti-rabbit IgG antibody (Serotec, Düsseldorf, Germany) for 1 h at RT. Proteins were then visualized with enhanced chemiluminescence (ECL) reagent on X-ray films (Euromed; Christiansen, Planegg, Germany). Remaining supernatant of immunoprecipitation was stored at −20°C for further processing.

### Identification of Co-purified Proteins by Liquid-chromatography Mass Spectrometry/mass Spectrometry (LC-MS/MS)

Directly prior to LC-MS/MS analysis, immunoprecipitated proteins were digested in trypsin and resulting peptides were separated on a reversed phase chromatography column (PepMap, 15 cm×75 µm ID, 3 µm/100A pore size, LC Packings) operated on a nano-HPLC apparatus (Ultimate 3000, Dionex GmbH, Idstein, Germany). The nano-HPLC was connected to a linear quadrupole ion trap-Orbitrap (LTQ Orbitrap XL) mass spectrometer (ThermoFisher, Bremen, Germany). The mass spectrometer was operated in the data-dependent mode to automatically switch between Orbitrap-MS and LTQ-MS/MS acquisition. Survey full scan MS spectra (from m/z 300 to 1500) were acquired in the Orbitrap resolution R = 60,000 at m/z 400. Up to ten most intense ions were in parallel selected for fragmentation on the linear ion trap using collision induced dissociation at a target value of 100,000 ions and subsequently dynamically excluded for 30 s. General mass spectrometry settings were: electrospray voltage, 1.25–1.4 kV; no sheath and auxiliary gas flow; ion selection threshold for MS/MS, 500 counts; activation Q-value for MS/MS, 0.25 and activation time for MS/MS, 30 ms. MS/MS spectra were exported from the Progenesis software as Mascot Generic file (mgf) and used for peptide identification using Mascot (Matrix Science, London, UK; http://www.matrixscience.com), the Uniprot database (http://www.uniprot.org) restricted to mammalian entries and the Ensembl cat database (http://www.ensembl.org) in particular. A protein was considered as identified if the confidence score was higher than 30 and if the significance threshold was p≤0.01. For quantification, all peptides allocated to a protein were included and the total cumulative abundance of the protein was calculated by summing the abundances of all peptides. Multiple interaction candidates of fibronectin were discovered of which peptide identifications are listed in table 1.

**Table 1:** Urine fibronectin co-purified proteins identified by mass spectrometry.

Protein name	Accession no.^a^	Peptide count^b^	Confidence score^c^	Maximum fold change^d^
Fibronectin	ENSFCAP00000008544	11	575	10.81
Ig kappa chain V region 3315	P01683	2	76	50166.98
Ig gamma chain C region	P01870	1	1489	6.25
Alpha-S1-casein	P02662	2	82	13.03
Caspase-14	P31944	1	51	7.44
Complement C4-A	P0C0L4	2	78	10.14
Galectin-7	P47929	2	104	23.95
Fatty acid-binding protein, intestinal	P12104	1	63	14.87
Thioredoxin	P10599	1	62	14.98

Multiple co-purified proteins of fibronectin could be identified in urine of FIC cases by LC-MS/MS of which eight are listed. a) Accession number as listed on Uniprot (http://www.uniprot.org) or Ensembl (http://www.ensembl.org) databases, b) Number of peptides the protein was identified with, c) Confidence score as given in Mascot were considered as significant if the value was higher than 30 (*p≤0.01), d) Ratio of control IP and FIC IP cumulated peptide intensity signal strengths (progenesis values).

### Verification and Quantification of Co-purified Proteins

#### SDS-PAGE, western blotting and signal quantification

For protein separation, SDS-PAGE was performed loading equal amounts of total protein from all urine supernatants followed by semidry blotting onto PVDF membranes (GE Healthcare). Unspecific binding was then blocked with 1% PVP-T for 1 h at RT. Blots were incubated overnight at 4°C with the according primary antibody. For detection of candidate proteins, polyclonal rabbit anti-human complement C4-A (C4a) antibody (Abcam, Berlin, Germany) was used at a working dilution of 1∶500. Rabbit polyclonal antibody against human galectin-7 (Abcam) was used at a dilution of 1∶3000. Polyclonal goat anti-human fatty acid-binding protein 2 (I-FABP) antibody (Abcam) was used at a dilution of 1∶1000 and rabbit polyclonal anti-human thioredoxin antibody (Abcam) was utilized at a working dilution of 1∶400. Polyclonal rabbit anti-human NF-κB antibody against subunit p65 was purchased from Cell Signaling, (Frankfurt (Main), Germany) and was used at a dilution of 1∶1000. Working dilution for polyclonal anti-human p38 MAPK antibody (Cell Signaling) was 1∶300. After three washing steps in PBS-T, blots were incubated in respective horseradish peroxidase-conjugated secondary antibody for 1 h at RT to detect binding of primary antibody. As secondary antibodies, goat anti-rabbit IgG (Serotec, Düsseldorf, Germany, dilution 1∶5000) or rabbit anti-goat IgG (Sigma-Aldrich, Deisenhofen, Germany; dilution 1∶1000) were utilized. Negative controls for all Western blot experiments included omission of the primary antibody as well as incubation with isotype-matched primary antibody of irrelevant specificity.

After twelve further washing steps in PBS-T, signals were detected by ECL on a radiographic film (Euromed). Western blots were imaged on a transmission scanner operated by LAB SCAN 5.0 software and Western blot signals were quantified by means of densitometry using ImageQuantTL software v2005 (all GE Healthcare).

#### Immunofluorescent labelling of target tissue

Urinary bladder tissue blocks were sectioned and subsequently mounted on coated slides (Superfrost; Menzel, Braunschweig, Germany). Heat antigen retrieval was processed at 99°C for 15 min in 0.1 M EDTA-NaOH buffer (pH 8.0). Tissue sections were blocked with 1% BSA in TBS-T and appropriate serum for 40 min at RT prior to incubation with primary antibody. Blocking serum was selected according to the species the secondary antibody was obtained from. In the case of labelling with multiple antibodies, blocking steps (ProteinBlock; Dako, Hamburg, Germany) were inserted between each antibody incubation. Tissue sections were fluorescently labelled by incubation with primary antibodies against fibronectin (monoclonal mouse anti-human fibronectin-antibody, 1∶100), C4a (1∶50), galectin-7 (1∶500), I-FABP (1∶200), thioredoxin (1∶100), NF-κB p65 (1∶50), p38 MAPK (1∶50) (all antibodies from Abcam) and mouse anti-human CD117 (1∶50; Serotec); at 4°C overnight followed by incubation with the respective secondary antibody for 30 min at RT. Secondary antibodies were Alexa Fluor dye-labelled and purchased from Invitrogen (Karlsruhe, Germany). All antibodies were used at a working dilution of 1∶500 (goat anti-rabbit IgG Alexa 647, donkey anti-goat IgG Alexa 546 and goat anti-mouse IgG Alexa 488). Isotype controls were included as negative controls in all immunohistochemical stainings. Cell nuclei were stained with 4′6-diamidino-2-phenylindol (Invitrogen; dilution 1∶1000). Finally, sections were mounted using fluorescence mounting medium. Fluorescent images were recorded with the Axio Imager M1 (Zeiss, Göttingen, Germany) and examined with Axio Vision 4.6 software (Zeiss).

#### Statistical analysis

Calculation of statistical significance was performed using the Paleontological Statistics (PAST) software (http://folk.uio.no/ohammer/past/index.html). Variance of protein expression quantified by means of densitometry using the ImageQuantTL software was analysed by a Kolmogorov-Smirnov test. Since the data were not distributed normally, the Mann-Whitney test was used to calculate statistical significance. The differences in the protein expression were considered as significant if the p-value was ≤0.05.

## Results

### Novel Potential Interacting Partners of Fibronectin Identified

For identification of the proteins that coprecipitated with fibronectin in the immunoprecipitation assay, LC-MS/MS analysis was used. Multiple co-purified proteins could be clearly identified as well as fibronectin itself, emphasising the affinity of the used antibody actually directed against a human target protein to the feline one (table 1). Peptides with a maximum fold change of more than 5.5 are listed in table 1. These identified proteins were Ig kappa chain region V 3315, Ig gamma chain C region, alpha-S1-casein, caspase-14, C4a, galectin-7, I-FABP and thioredoxin. Next, we decided to verify changed expression patterns of the latter four candidates in urine of a cohort of healthy and FIC cases.

### Expression of Candidates in Urine of FIC Diseased Cases and Healthy Controls

#### Complement C4a and galectin-7 levels are increased in urine of FIC cases

**Figure 1:**
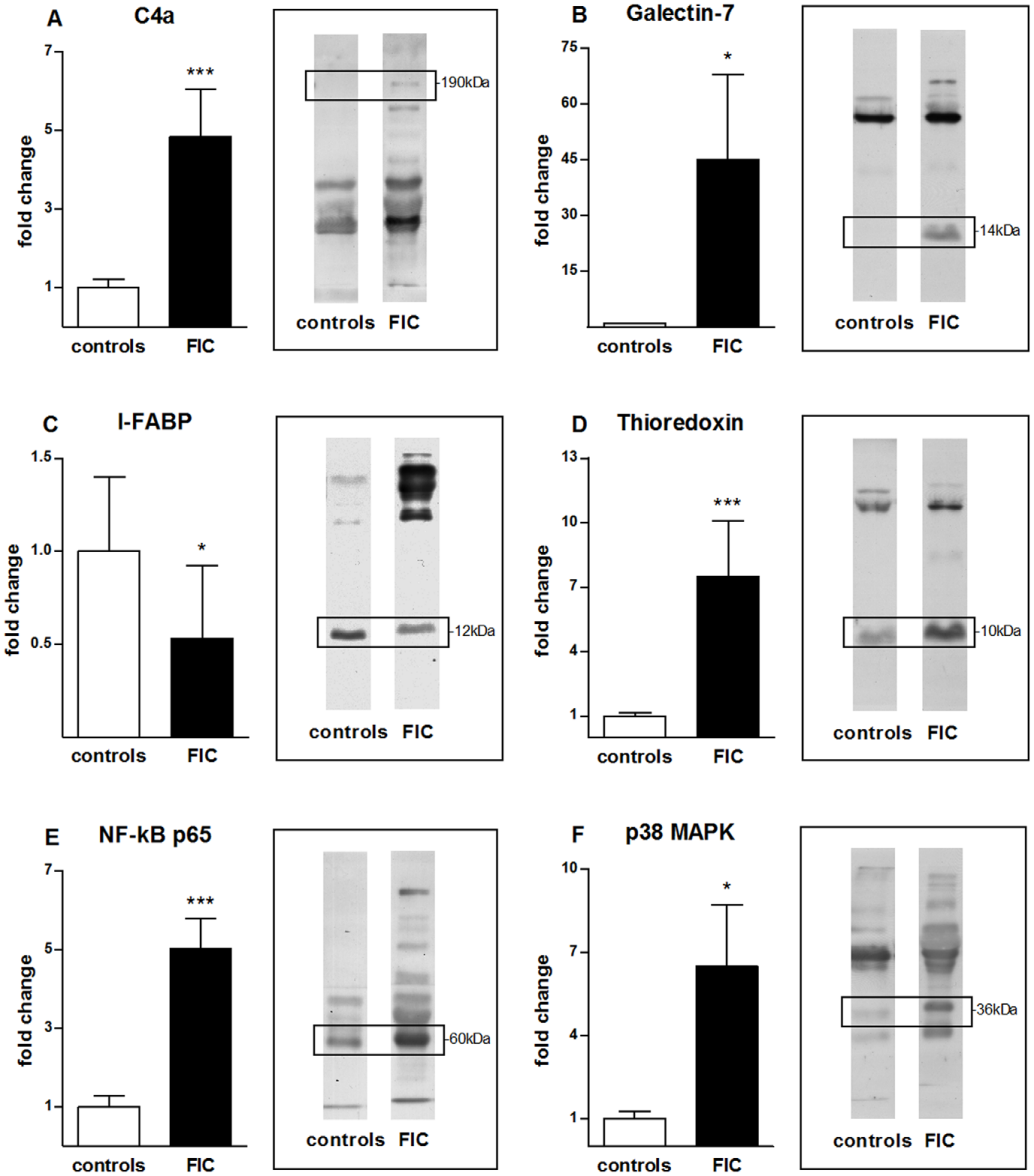
Quantification of signal intensities of co-purified candidate proteins of healthy and FIC diseased cases. Western blot signal intensity of healthy controls (white columns, left, n = 20) and diseased urine samples (grey columns, right, n = 16) were compared for the following co-purified proteins: Complement component 4a (C4a) (A), galectin-7 (B), fatty acid-binding protein, intestinal (I-FABP) (C), thioredoxin (D), NF-κB p65 (E) and p38 MAPK (F). The according Western blot strips visualize the quantitative difference of signal intensities. The left strips show representative control blots, the right ones blots with FIC urine. The corresponding band sizes are displayed in black boxes. Signals were quantified by densitometry and statistical significance was calculated using the Mann-Whitney test. Data are represented in a column bar graph as means with SEM. Expression level of C4a (A) shows a significant (***p≤0.001) increase in urine of FIC diseased cases with an almost 5-fold higher concentration compared to urine of healthy controls. Galectin-7 (B) is significantly (*p≤0.05) upregulated in FIC diseased samples compared to healthy control samples with a 45-fold higher expression in FIC affected cases. Quantification of I-FABP expression (C) results in a significant (*p≤0.05) decrease in urine of FIC cases compared to urine of healthy controls. Thioredoxin (D) is significantly (***p≤0.001) increased by a factor of 7.5 in FIC compared to urine of controls. Abundances of NF-kB p65 (E) significantly (***p≤0.001) increase in diseased specimens compared to healthy specimens with a 5-fold higher expression just as p38 MAPK (F) showing an 6.5-fold higher expression (*p≤0.05) in FIC diseased urines in comparison to healthy control urines.

Quantification of C4a signal intensities in urine of FIC cases compared to healthy controls showed a significant (p≤0.001) increase of C4a levels in the majority of tested urine samples of FIC cases (Fig. 1A, black column) with an almost 5-fold higher concentration in FIC urines compared to healthy control urines (Fig. 1A, white column).

In urine of FIC cases, (Fig. 1B, black column) we found an extraordinary upregulation of galectin-7 with a 45-fold higher expression in urine of FIC compared to healthy control urine (Fig. 1B, white column) with a p-value of ≤0.05. Interestingly, only some urine samples of FIC affected cases revealed a higher expression, whereas the signal intensity of galectin-7 in some FIC and all healthy cases was negative (Fig. 1B).

#### I-FABP level is decreased in urine of FIC cases

Fatty acid-binding protein 2 (I-FABP), belongs to the fatty acid-binding protein family and is generally expressed in the entirety of the intestine [18]. I-FABP is physiologically expressed in urine of healthy cats as well (Fig. 1C, white column). In contrast, we found a significant decrease of I-FABP levels to only one half of the physiological amount in urine of FIC diseased cases, indicating a loss of I-FABP in FIC (Fig. 1C, black column).

#### Thioredoxin and the signal transduction molecules NF-κB p65 and p38 MAPK are upregulated in FIC diseased urine

Thioredoxin is a small redox-regulating protein that belongs to the thioredoxin family and plays a role in a wide variety of biological functions e.g. in oxidative stress [19]. We quantified the signal intensity of thioredoxin in urine of healthy (Fig. 1D, white column) and FIC diseased cases (Fig. 1D, black column). An average increase by a factor of 7.5 in the majority of FIC samples compared to healthy control samples could be observed. Since thioredoxin was significantly upregulated in urine of FIC affected cases, we next were interested which signal transduction pathways were changed in disease. Therefore, we examined the downstream molecules NF-κB p65 (NF-κB pathway) and p38 MAPK (MAPK pathway) [20]–[21]. Interestingly, we found a 5-fold increased concentration of NF-κB p65 in almost every urine sample of FIC affected cases (Fig. 1E, black column) compared to healthy control urines (Fig. 1E, white column). Moreover, quantification of p38 MAPK expression showed an average upregulation by factor 6.5 in FIC urine (Fig. 1F, black column) in contrast to physiological p38 MAPK concentration in healthy control urine (Fig. 1F, white column).

### Expression of Fibronectin and its Potential Interacting Partners in Target Tissue of FIC Cases and Healthy Controls

**Figure 2:**
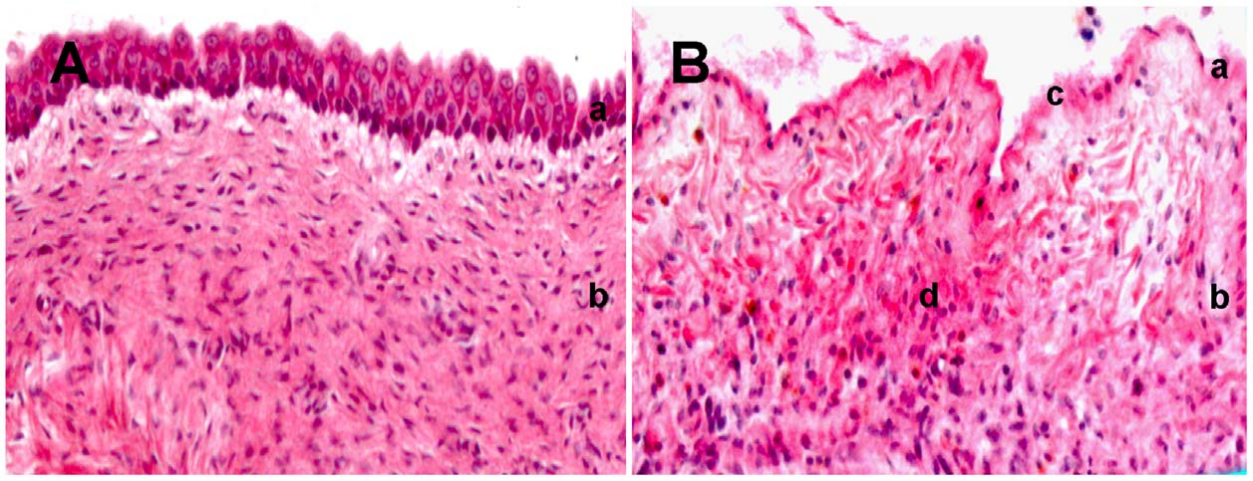
H&E staining of normal feline bladder (A) and FIC diseased bladder (B). Histological sections stained with Haematoxylin and Eosin. Healthy urinary bladder section (A) shows characteristic architecture compared with FIC diseased bladder tissue (B), where a loss of normal bladder wall physiology can be seen. Notice the marked loss of transitional cell epithelium, the intramucosal bleeding and oedema in the FIC section (B). a = Transitional cell epithelium, b = Lamina propria mucosae, c = Loss of transitional cell epithelium, d = Intramucosal bleeding and oedema.

**Figure 3:**
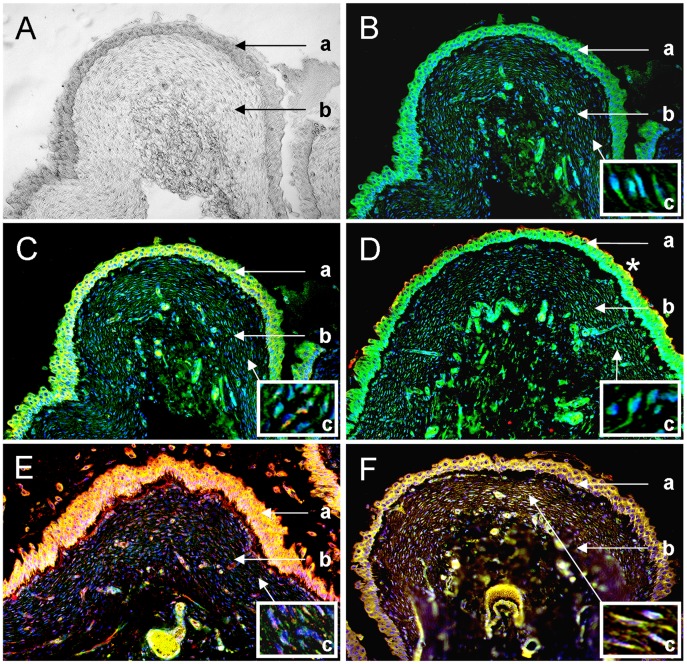
Expression of co-purified proteins in healthy bladder tissue. Immunohistochemical double labelling of CD117 and co-purified proteins in a representative healthy bladder tissue. DIC image of healthy bladder tissue (A). CD117 (green) shows a marked reactivity in the epithelial cells of the urothel and in the interstitial cells of the lamina propria of the healthy bladder (B). Overlay image of C4a (red) and CD117 (green) reveals considerable co-localization (overlapping results in yellow colour) at the cell nuclei of the urothelial cells and a scattered expression in the interstitial cells of the lamina propria (C). Galectin-7 (red) and CD117 (green) show a co-localization in the umbrella cells (marked with an asterisk) of the transitional cell epithelium, whereas reactivity of both proteins in the epithelial cells of the urothel indicate a co-expression. Cells of the lamina propria are only CD117 positive (D). I-FABP (red) and CD117 (green) overlay is visible only in the interstitial cells of the lamina propria. Additionally, I-FABP reactivity is seen extracellularly and is distinctly expressed in the basal membrane (E). Thioredoxin (red) and CD117 (green) co-localize distinctly at all cell nuclei of the transitional epithelial cells and in the interstitial cells of the subepithelial tunic (F). The blue colour reveals staining of cell nuclei (DAPI). a = Transitional cell epithelium, b = Lamina propria mucosae, c = Inserted box shows magnification of respective cells in the lamina propria mucosae.

**Figure 4:**
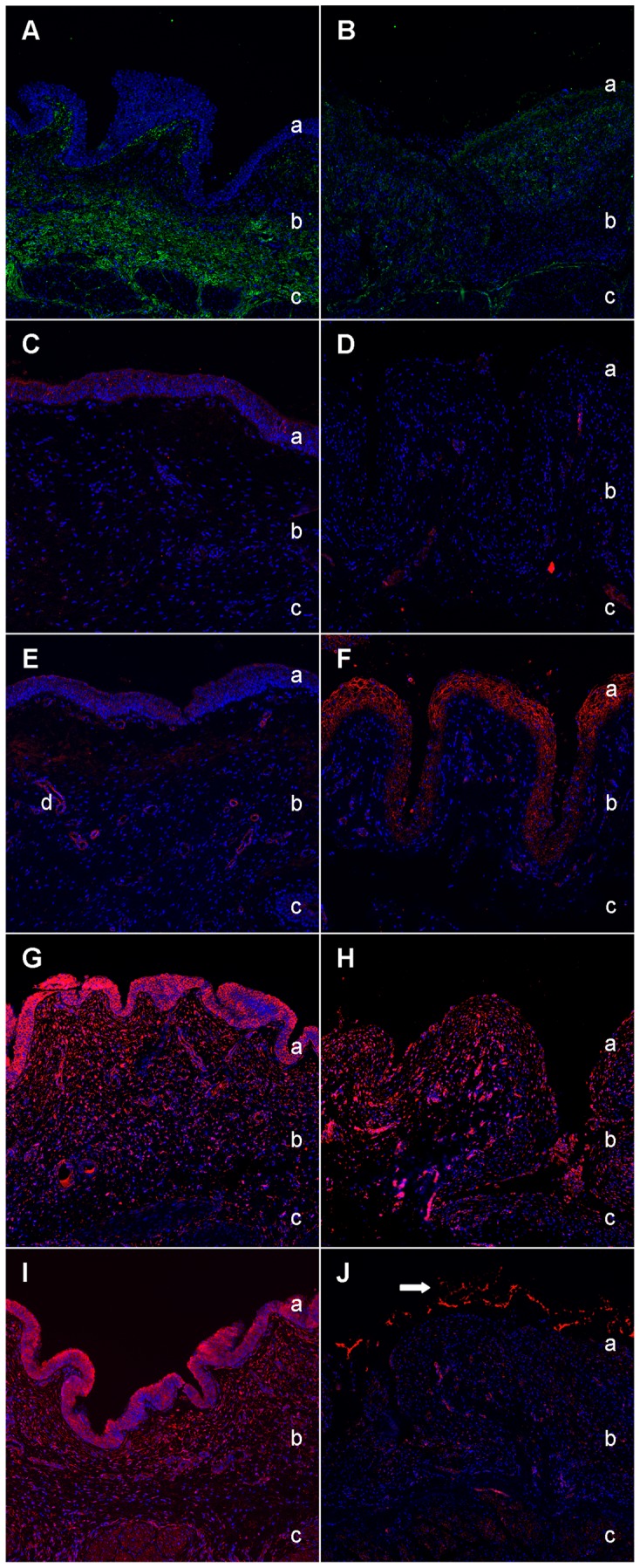
Expression pattern of fibronectin and its co-purified proteins in healthy and diseased bladder tissue. Urinary bladder expression of fibronectin (green) and its co-purified proteins (red) in a representative healthy (left panels) and FIC diseased bladder (right panels). Physiological distribution of fibronectin (green) in healthy bladder (A). Extracellular matrix of the lamina propria mucosae and the muscle tunic show a distinct immunoreactivity for fibronectin, whereas a loss in FIC affected bladder tissue (B), especially in the subepithelial and muscular tunices, is evident. C4a (red) is moderately expressed in the apical transitional cell epithelium of the physiological bladder (C) and disappears in the FIC affected bladder tissue (D). Galectin-7 (red) is expressed especially in umbrella cells of the transitional epithelium and around blood vessels in the lamina propria under normal condition (E). In contrast, expression changes profoundly in FIC affected bladders to distinct expression in the transitional cell epithelium (F). Reactivity of I-FABP (red) throughout all tunices in healthy bladder (G) almost disappears in FIC diseased tissue (H). Thioredoxin (red) reveals a predominant signal in the entire healthy bladder tissue (I) compared to a leakage of thioredoxin into the lumen (arrow) of FIC affected bladder tissue resulting in a slight immunoreactivity of the diseased bladder tissue (J). The blue colour reveals staining of cell nuclei (DAPI). a = Transitional cell epithelium, b = Lamina propria mucosae, c = Muscle tunic, d = Normal vessel.

To examine the physiological expression of identified co-purified proteins of fibronectin, we investigated candidate expression patterns with immunohistochemical methods (Fig. 2A, H&E staining, Fig. 3, fluorescent double staining and Fig. 4, left panel). Then, we analyzed appearance of protein partners under FIC condition (Fig. 2B, H&E staining, Fig. 4 right panel). Representative staining of FIC affected bladder tissue with H&E showed marked destruction of normal bladder wall physiology (Fig. 2A) compared to the characteristic architecture of healthy bladder tissue (Fig. 2B).

#### I-FABP and thioredoxin co-localize with the interstitial cell marker CD117 in the lamina propria mucosae

In order to define the specific localization of all candidates under normal condition and to demonstrate the association to certain structures of the bladder tissue (Fig. 3A), we performed immunohistochemical double staining of co-purified proteins with interstitial cell marker CD117, which was markedly expressed in the umbrella and epithelial cells of the transitional cell epithelium as well as in the interstitial cells of the lamina propria mucosae (Fig. 3B). C4a and CD117 overlay could be observed in the urothelial cells as well as a scattered expression in the interstitial cells of the subepithelial layer (Fig. 3C). Galectin-7 only showed an overlay with CD117 in the umbrella cells of the transitional cell epithelium of the healthy bladder and co-localized in the urothelial cells (Fig. 3D). Besides a clear overlay of I-FABP and CD117 in the transitional cell epithelium, an additional co-localization in the interstitial cells of the lamina propria and a separate expression of I-FABP extracellularly could be seen (Fig. 3E). Thioredoxin expression overlapped with CD117 in the epithelial cells of the urothel as well as in the interstitial cells of the lamina propria (Fig. 3F).

#### C4a expression decreases whereas galectin-7 shows a distinct increase of signal intensity in the transitional cell epithelium of FIC cases

In comparison to physiological fibronectin expression in healthy bladder tissues (Fig. 4A) and loss of fibronectin in FIC diseased tissues (Fig. 4B), C4a was primarily associated to the apical transitional cell epithelium in physiological condition (Fig. 4C), whereas, similar to fibronectin, an absence of C4a expression from bladders of FIC cases was detected (Fig. 4D). Galectin-7 showed a considerable change in the expression of healthy bladder tissues (Fig. 4E) compared to FIC affected tissues (Fig. 4F): a slight signal intensity in the transitional cell epithelium and around blood vessels in the subepithelial tunic of healthy urinary bladders (Fig. 4E), with an obvious increase of galectin-7 expression in FIC diseased bladder tissues in the cytoplasm of cells of the transitional epithelium (Fig. 4F).

#### I-FABP and thioredoxin are both expressed in the urinary bladder tissue of healthy controls whereas thioredoxin shows a distinct loss of signal intensity in FIC diseased bladders

I-FABP was expressed throughout all layers of healthy urinary bladder tissues with a clear reactivity in the transitional cell epithelium (Fig. 4G). In FIC affected tissues (Fig. 4H), loss of signal intensity was visible in all tunices of the urinary bladders.

Thioredoxin was detected in even amounts in all tunices of healthy bladders (Fig. 4I). In FIC cases (Fig. 4J), all tunices of the urinary bladders showed a decrease of reactivity of thioredoxin with a marked leakage into the bladder lumen.

#### Fibronectin and its interactor thioredoxin are colocalized in the subepithelial and muscular tunices of healthy bladder tissues

**Figure 5:**
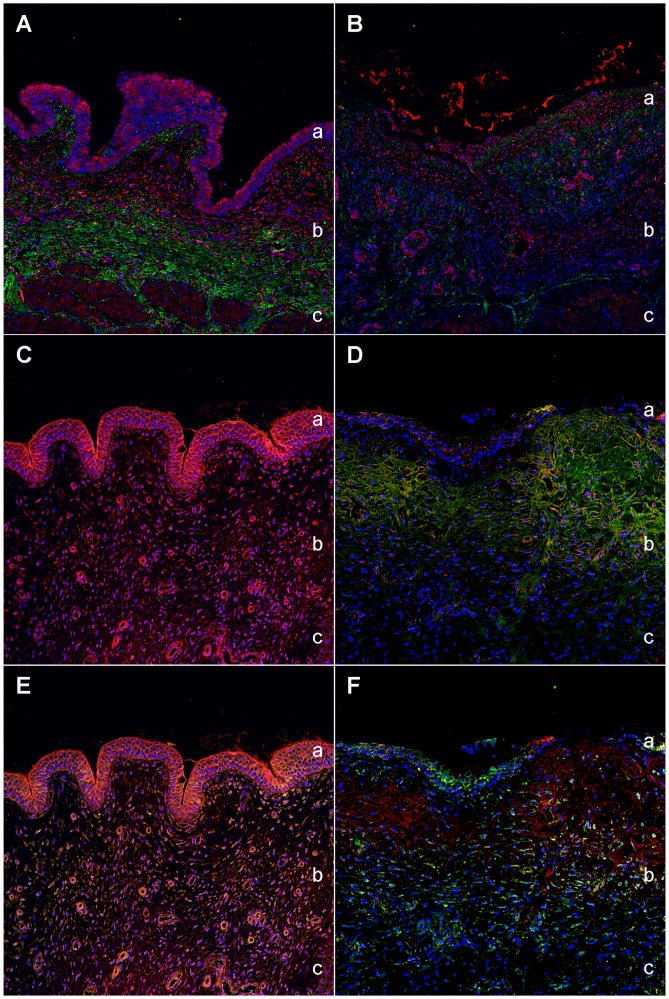
Immunohistochemical double labelling of candidates in healthy (left panels) and diseased bladder tissue (right panels). Immunohistochemical double staining of a healthy urinary bladder (A) shows considerable colocalization of fibronectin (green) and its interactor thioredoxin (red) in the subepithelial and muscular tunices. In contrast, lack of green and red colour is evident in FIC (B), indicating a loss of both fibronectin and thioredoxin from its normal distribution in healthy bladder tissue. Overlay image of thioredoxin (red) and NF-κB p65 (green) in a healthy bladder tissue (C) shows a predominant signal of thioredoxin in all tunices of the bladder, whereas NF-κB p65 is not detectable in any tunic of the healthy bladder. In contrast, thioredoxin and NF-κB p65 colocalize (overlapping results in yellow colour) in the lamina propria mucosae with the highest expression in the extracellular matrix of FIC diseased bladder tissue (D). P38 MAPK signal is of moderate intensity localized in transitional epithelium cells and around few blood vessels in the healthy bladder tissue (E). Note that the signal is exclusively of yellow colour indicating a colocalization with thioredoxin, whereas a green colour signal is not visible at all. In contrast, p38 MAPK (green) was highly expressed in the cytoplasm of umbrella cells of the transitional cell epithelium as well as a scattered expression around cell nuclei in the subepithelial and muscular tunices of FIC diseased bladder sections without distinct colocalization (F). The blue colour reveals staining of cell nuclei (DAPI). a = Transitional cell epithelium, b = Lamina propria mucosae, c = Muscle tunic.

A coexpression for fibronectin and thioredoxin could be demonstrated in the lamina propria mucosae and the muscle tunic in healthy urinary bladders (Fig. 5A). Additionally, fibronectin was mainly present in the extracellular matrix (ECM), whereas thioredoxin could be found in the cytoplasm of cells. We also observed a distinct leakage of signal intensity of both molecules in all layers of FIC diseased tissues (Fig. 5B) with a loss of coexpression in the subepithelial and muscular tunices.

#### NF-κB p65 and p38 MAPK clearly appear in FIC affected tissues

Quantification of Western blot analyses showed a distinct upregulation of thioredoxin as well as the signal transduction molecules NF-κB p65 and p38 MAPK in urine of FIC cases (Fig. 1E and 1F). We performed immunohistochemical double labelling of thioredoxin and either NF-κB p65 or p38 MAPK to investigate the connection between thioredoxin and related signal transduction cascades in urinary bladder tissues of healthy and diseased cases. Although thioredoxin was expressed in all tunices of the healthy urinary bladders (Fig. 5C), NF-κB p65 was not detectable in any layer of healthy bladder. Interestingly, thioredoxin was almost absent in FIC diseased sections (Fig. 5D) except for a weak signal in the transitional cell epithelium and the lamina propria of the bladder tissues. In contrast, NF-κB p65 was clearly expressed in the subepithelial tunic of FIC bladders. Interestingly, a focal colocalization of NF-κB p65 and thioredoxin could be seen in the lamina propria of FIC diseased bladder tissues at NF-κB p65 expression sites. P38 MAPK showed a slight expression in healthy bladder tissues in the transitional cell epithelium and the lamina propria mucosae (Fig. 5E). In FIC affected tissues, p38 MAPK was clearly expressed in the cytoplasm of the umbrella cells of the transitional cell epithelium as well as in a scattered pattern around cell nuclei in the subepithelial and muscular tunices, without a co-localization (Fig. 5F).

## Discussion

IC/painful bladder syndrome is a common human disease with a burdensome character that leads to an adverse impact on quality of life for affected people [22]. The only spontaneous animal model for IC in humans is currently the feline type of urinary tract disorder. Besides similarities in the clinical appearance and the spontaneous occurrence of both diseases, there are many comparable pathological alterations that indicate the high transferability and relevant input of FIC and IC research [23]. Regarding this, we focussed our study on the protein interaction network of fibronectin in FIC diseased cases to elucidate possible pathomechanisms in the development of this disorder.

To identify potential interacting partners of fibronectin in diseased urine, we performed co-immunoprecipitation followed by mass spectrometry analysis. This approach was successful and numerous co-purified proteins of fibronectin could be discovered (Table 1). We closely examined four candidates of the co-purified proteins and verified their expression patterns in urine of a cohort of healthy and FIC cases (Fig. 1). Furthermore, we investigated their physiological expression and specific localization in relation to interstitial cell markers in healthy bladder tissues and their expression patterns under FIC condition with immunohistochemical methods (Fig. 3 and 4).

A candidate closely examined was C4a, a member of the complement cascade [24]. Several studies previously investigated the involvement of complement in the pathogenesis of human IC [25]–[27]. A significant depletion of C4 in serum of IC patients could be found suggesting an involvement of a chronic local immunological process in the pathogenesis of this disease [25]. Higher amounts of urinary C4a in FIC cases could be the result of a significant increase of serum levels in FIC. However, fibronectin was recently reported to be increased in the urine of FIC cats due to leakage from damaged urinary bladder tissue [15]. Therefore, the observed decreased abundance of C4a in FIC affected bladder tissue (Fig. 4C and D), but increased abundance in urine of diseased cases (Fig. 1A) could both be resulting from cell death and tissue damage. Helin et al. elucidated the impact of complement to the development of tissue injury and the chronic self-perpetuating inflammation typical for IC [27]. Under physiological conditions complement activation is well-controlled, whereas pathological alteration accelerates its activation due to stimuli such as tissue injury [28]. For this reason, we presume that in the case of FIC, tissue damage of affected bladders cause an augmented activation of the complement system and an increased abundance of peptide mediators like C4a in the inflammatory process, which in return leak into the urine through the damaged, hyper-permeable urinary bladder wall. Furthermore, intense C4a activation could generate a more excessive inflammatory response than necessary to eliminate underlying damage and therefore play a role in the chronic and relapsing character of the disease.

A very interesting co-purified protein of fibronectin identified in this study is galectin-7, which was only present in urine of FIC cases but not in control urine (Fig. 1B). Galectin-7 is mainly distributed in stratified squamous epithelium in various tissues and its functions include cell-to-cell adhesion, cell-matrix interaction, growth regulation and apoptosis [29]. In this study, a physiological presence of galectin-7 in the transitional epithelium and around blood vessels in the subepithelial tunic could be demonstrated in healthy bladder tissues where it co-localizes with CD117, a protein expressed by interstitial cells of Cajal in the transitional cell epithelium of the lower urinary tract (Fig. 3D and 4E) [30]. Interestingly, FIC diseased bladder tissues showed an increase of galectin-7 signal intensity in the transitional epithelium (Fig. 4F). Galectin-7 plays a crucial role in reepithelialisation of corneal [31], epidermal wounds [32] and in wound repair of polarized kidney cells [33]. An increased abundance in the transitional cell epithelium of FIC diseased bladders indicates an upregulation of this protein due to loss of physiological structure of the bladder [15]. We believe that galectin-7 plays an important role in wound healing and reepithelialisation of the impaired tissue in FIC cases as well. Furthermore, the extent of acceleration of the reepithelialisation of galectin-7 in corneal wounds was greater than that of growth factors [31]. Moreover, the clinical potential of galectin-7 seems to be more attractive than that of growth factors due to absent cell mitosis in epithelial cells [34]. On this account, galectin-7 could be of greatest interest for developing novel therapeutic strategies for treatment of FIC and thus also IC.

Fibrosis has recently been proposed to play an important role in the pathogenesis of FIC [15]. Furthermore, the primary cause of fibrotic disease has been suggested to be an uncontrolled differentiation of fibroblasts into myofibroblasts [35]. A novel study investigated the impact of galectins on the formation of the ECM demonstrating a galectin-7 dependent stimulation of myofibroblast formation and a marked production of a three-dimensional network of fibers containing fibronectin [36]. These findings provide an interesting insight into the pathogenesis of disorders engraved by their fibrotic character such as FIC and is consistent with the findings of our study. In this context, galectin-7 could serve as a positive regulator of tissue fibrosis preventing uncontrolled ECM formation as a result of chronically relapsing inflammation in FIC affected tissue.

A further protein that we identified as a possible binding partner of fibronectin is I-FABP. Several studies described the beneficial use of I-FABP as a urinary marker for intestinal injuries such as during or after acute ischemic diseases [37]–[38] as well as urothelial carcinomas of the upper urinary tract [39]. We found a reduction of I-FABP in urine of FIC diseased cases by 50% compared to the physiological amount in healthy (Fig. 1C). We furthermore demonstrated a high abundance of I-FABP in all layers of the bladders, especially in the transitional epithelium (Fig. 4G) and a lack of I-FABP in FIC tissues (Fig. 4H) as well as a correlation of I-FABP to interstitial cells of the lamina propria (Fig. 3E). A loss of I-FABP in transitional epithelium of diseased tissues might be the result of an absence of cellular tissue. However, I-FABP concentration was also decreased in urine of FIC cases. Interestingly, Halldén et al. reported that I-FABP expression in intestinal epithelial cells is regulated by factors present in the extracellular matrix such as fibronectin [40]. The decreased concentration of fibronectin in the bladder tissue due to an increased bladder permeability [15] could thus be the trigger for downregulation of I-FABP expression. Since the exact pathways are still unclear, further studies are necessary to elucidate I-FABP function in urinary bladder tissue as well.

Another candidate protein identified by mass spectrometry that seems to be of great significance is thioredoxin. Thioredoxin is important for many biological functions, such as defense against oxidative stress and regulation of apoptosis [41]. In this study, a significantly higher concentration of thioredoxin in urine of FIC affected cats compared to urine of healthy controls could be demonstrated (Fig. 1D). We also verified thioredoxin expression in the transitional cell epithelium, especially in umbrella cells, of healthy urinary bladder tissue (Fig. 3F). Increased abundance of thioredoxin in response to oxidative stress and a protective role of thioredoxin were already reported in renal ischemia/reperfusion injury inducing secretion of thioredoxin into the urine [42]. The authors suggested an excretion that is not due to leakage from dead cells since total protein levels were unchanged in the urine after reperfusion [42]. Thus, higher amounts of thioredoxin in urine of FIC cases could be caused by hypoxia in the kidneys as a result of obstruction of the lower urinary tract. However, distinct immunohistochemical staining of thioredoxin in control tissues (Fig. 4I) and a loss of signal intensity in all tunices of diseased bladders (Fig. 4J) argue against this hypothesis. Thioredoxin was shown to be over-expressed in bladders of urinary outlet obstructed rats [43]. Furthermore, a recent study experimentally induced IC in rats subsequent to exposure to oxidative stress using bladder instillation of a nitric oxide donor gel [44]. We therefore assume that the urinary bladder of FIC diseased cases is subject to apoptosis. As a consequence, injured tissue cells could secrete cytoplasmic thioredoxin into the urine where it operates as protector against oxidative stress. In accordance to previous reports, these findings are suggesting a protective role of extracellularly injected recombinant human thioredoxin on injury, for example in the case of neuronal cells induced by ischemia/reperfusion [45]. Thioredoxin may therefore be a promising candidate for therapeutics to improve the prognosis and development of FIC as well as of its human counterpart, IC.

To understand the relationship between fibronectin and its potential interacting proteins, we performed immunohistochemical double labelling to determine the expression patterns of fibronectin in association with thioredoxin. We could demonstrate a colocalization of fibronectin and thioredoxin in the subepithelial and muscular tunices of the healthy bladder (Fig. 5A), whereas colocalization disappeared in FIC tissues (Fig. 5B). Interestingly, a previous study investigating the effect of thioredoxin reductase 1 (TrxR1) silencing on gene expression in HepG2 cells identified a regulation of fibronectin 1 gene [46]. However, to which extent the interaction of fibronectin and thioredoxin takes place is still unclear.

Regarding biological functions of thioredoxin, a further important role is the redox regulation of transcription factors such as NF-κB [41]. Immunohistochemical localization experiments of NF-κB in bladder biopsies from patients with IC showed a predominant activation in bladder urothelial cells and cells of the submucosal layer in biopsies from patients with IC compared to a diffuse and faint staining in control samples [47]. These findings are consistent with our colocalization results of thioredoxin and NF-κB in bladder tissue of healthy (Fig. 5C) and FIC cases (Fig. 5D). In addition, we found a significantly higher concentration of NF-κB in urine of FIC cases (Fig. 1E). Research on NF-κB-dependent processes in the pathogenesis of IC revealed an interesting NF-κB-regulated increase of proinflammatory cytokine gene products in the urine of IC patients in comparison to controls suggesting a perpetuation of NF-κB activation via a positive regulatory loop [48]. We could demonstrate an interaction of thioredoxin and NF-κB in FIC tissues which is supposed to play a crucial role in the pathogenesis of this disease. NF-κB activation could reinforce proinflammatory cytokine expression in the development of FIC. In turn, NF-κB-regulated circulation of proinflammatory factors in combination with an increased concentration of thioredoxin could therefore reinforce NF-κB stimulation. This pathway could pose a vicious circle in the pathogenesis of the disease and could lead to a chronic inflammatory response underlying the relapsing nature of FIC.

Another signal transduction pathway we were interested in is the p38 MAPK pathway. Previous studies identified thioredoxin as a negative regulator of the p38 MAPK pathway, which plays a role in apoptosis regulation [21], [49]. Moreover, p38 MAPK is well known to be upregulated in urinary bladder cancer cells playing a crucial role in tumour growth and progression [50]. In our study, comparison of the concentration of urinary p38 MAPK in healthy and FIC diseased specimens revealed a 6.5-fold higher concentration in diseased urine (Fig. 1F). Furthermore, we could demonstrate that in contrast to the low expression of p38 MAPK in healthy tissues in transitional epithelium and subepithelial tunices (Fig. 5E), p38 MAPK showed considerably higher expression in FIC tissues (Fig. 5F). Interestingly, immunohistochemical double staining of thioredoxin and p38 MAPK revealed almost no colocalization in FIC affected bladder tissues. This could be resulting from an inhibitory effect of thioredoxin expression on p38 MAPK under FIC conditions interfering with cytokine- and stress-induced apoptosis. However, loss of thioredoxin into the urine could exhibit a negative factor in the progression of the disease.

In conclusion, we identified different co-purified proteins of fibronectin that are present in urine and urinary bladder tissue of healthy controls and FIC cases. We could demonstrate a significant alteration in diseased conditions compared to healthy controls indicating an important role of these possible interacting partners in the pathomechanism of the disease. As FIC serves as spontaneous animal model for human IC, our findings could also provide an interesting insight into the pathogenesis of IC. Additionally, our study revealed an altered regulation of signal transduction pathways such as NF-κB and p38 MAPK in FIC. These pathways should be of major interest for future studies and might provide the basis for a novel approach in FIC therapy.
